# Frailty and clinical outcomes following aortic valve replacement

**DOI:** 10.1111/jocs.16801

**Published:** 2022-08-03

**Authors:** Eilon Ram, Yael Peled, Tali B. Miller, Efrat M. Dray, Ehud Karni, Ehud Raanani, Leonid Sternik

**Affiliations:** ^1^ Department of Cardiac Surgery Sheba Medical Center, Tel Hashomer, Affiliated to the Sackler School of Medicine Tel Aviv University Tel Aviv Israel; ^2^ Department of Cardiology, Sheba Medical Center, Tel Hashomer, Affiliated to the Sackler School of Medicine Tel Aviv University Tel Aviv Israel; ^3^ The Sheba Talpiot Medical Leadership Program Ramat Gan Israel

**Keywords:** aortic valve replacement, frailty, Norton score, risk stratification tool

## Abstract

**Background and Aims:**

The Norton score is a well‐known scale to assess frailty. Frailty and a low Norton score are associated with complications and mortality in hospitalized patients. We aimed to evaluate whether a low Norton score is associated with surgical complications and death after aortic valve replacement (AVR).

**Methods:**

From 2004 through 2020, we performed an observational study in a large tertiary medical center, which included all patients who had undergone isolated AVR surgery. Of the 1469 study patients, 618 patients (42%) had a low (<18) and 851 patients (58%) a high Norton score (≥18).

**Results:**

Frailer patients with a low Norton score had higher in‐hospital mortality compared to those with a high Norton score (5.5% vs. 0.8%, *p* < .001). The Norton score was significantly higher among patients who survived compared to those who died (17.5 ± 2.4 vs. 11.5 ± 5.2, *p* < .001). A low Norton score was associated with a threefold increased risk of in‐hospital mortality (odds ratio 3.03; 95% confidence interval [CI] 1.14–0.09, *p* = .034). Ten‐year mortality rate was higher among frailer patients with a low compared with a high Norton score (25.9%, 13.3%; hazard ratio 0.69, CI 0.48‐0.82, *p* < .001). By adding a Norton score to standard prognostic factors (age, gender, comorbidities, left ventricular ejection fraction, functional class) we showed a significant improvement of 59.4% (*p* < .001) for predicting 1‐year mortality, and 40.6% (*p* < .001) for predicting 10‐year mortality.

**Conclusions:**

Our findings show that the admission Norton score is a powerful marker of short‐ and long‐term mortality, and, therefore, should be considered as a risk stratification tool in patients who are candidates for AVR.

## 1 INTRODUCTION

Aortic valve stenosis (AS), the most commonly acquired valve disorder, is emerging as a new epidemic in the western world due to ageing populations.^1^ Surgical aortic valve replacement (AVR) and transcatheter aortic valve implantation (TAVI) are the only effective treatments for severe AV stenosis.

During the last decade, a growing interest has emerged in the assessment of frailty as an overall marker of functional impairment, and cognitive and nutritional status, thus playing a pivotal role in defining a patient's potential for recovery following AVR or TAVI. The prevalence of frailty ranges from 10% to 60%, depending on the cardiovascular burden of the population, as well as the specific frailty measures and thresholds utilized for its assessment.^2^, ^3^ The assessment of frailty should not rely on a subjective approach, such as the “eyeball test,” but rather on a combination of different objective estimates.

The Norton scoring system is a well‐known scale that includes five domains that concern fundamental aspects of well‐being: physical condition, mental state, activity, mobility, and continence. Previous publications have shown that low admission Norton scores are associated with complications and in‐hospital mortality in patients following different procedures and hospitalized patients in general.^4^, ^5^, ^6^

There is a lack of clinical data regarding the predictive value of the Norton score at admission on outcomes of patients undergoing AVR. In this study, we sought to evaluate whether a low Norton score is associated with surgical complications, prolonged hospitalization, and death after AVR.

## 2 METHODS

### 2.1 Study design and population

We performed a retrospective, observational study that included prospectively collected data from a large tertiary university hospital. Between January 2004 and August 2020, a total of 1469 patients underwent their first isolated AVR. All patients had a complete physical examination by a physician on admission and underwent a complete blood test, which was analyzed at the center's laboratory. Past medical history and current medications were all keyed into an electronic database. All patients were assessed by the department's nursing staff and were graded according to Norton parameters (Supporting Information: Table S1) upon admission before surgery.

The cohort was divided into two groups according to the admission Norton score: the high‐risk frailer patients had a low Norton score (<18), and the low‐risk patients had a high Norton score (≥18) according to the median Norton prognostic scaling score value. In a secondary analysis, the Norton score was assessed as a continuous measure.

The study was approved by the Sheba Medical Center Institutional Ethics Committee (Protocol no 4257). The requirement for informed consent was waived because of the retrospective nature of the study.

### 2.2 Surgical procedures and postoperative care

Standard cardiopulmonary bypass was established by cannulation of the ascending aorta and the right atrium or the femoral artery and vein in reoperation cases. Myocardial protection was achieved by using antegrade and/or retrograde cold blood cardioplegia.

After surgery, all patients were admitted to the intensive care unit (ICU) directly from the operating room. Following discharge from the ICU, patients were transferred either to a step‐down unit or directly to the floor, from where they were discharged either to their home or to a rehabilitation facility.

### 2.3 Data collection and follow‐up

All hospital data were ascertained by a hospital chart review. Data included: demographic parameters, medical history, chronic and periprocedural medical treatment, echocardiography measurements, procedural information, and outcome measures. Mortality data were ascertained from the Israeli Ministry of Interior Population Register through September 2020.

### 2.4 Statistical analysis

Baseline characteristics, comorbidities, echocardiographic parameters, and outcomes were compared according to the Norton score at admission. Data are presented as mean ± standard deviation. Continuous variables were tested with the Kolmogorov–Smirnov test for normal distribution. Categorical variables are given as frequencies and percentages. A *χ* ^2^ test was used for comparison of categorical variables between the low‐ and high‐Norton score groups. A Student's *t*‐test was performed for comparison of normally distributed continuous variables and Mann–Whitney *U* test for non‐normal distribution.

Multivariable logistic regression analysis was used to identify factors relating to in‐hospital mortality. All statistically different variables (*p* < .1) in the univariable analysis were entered into the model. The variables included by this indication were age, peripheral vascular disease, atrial fibrillation, chronic obstructive pulmonary disease (COPD), renal impairment, prior stroke, left ventricle ejection fraction, hemoglobin level at admission, and the Norton score. Gender (prespecified) was also included in the model due to its clinical importance. Survival analysis was performed using the Kaplan–Meier method, and comparison by the two groups was tested using the log‐rank test. Furthermore, mortality was evaluated by an additional model for the overall study period based on Cox regression, to estimate the average hazard ratio (HR). Statistically significant variables by univariable analysis and prespecified variables were used in the multivariable model to identify independent predictors of 10‐year mortality. The variables included in the final model were age, hypertension, peripheral vascular disease, diabetes, renal impairment, pulmonary hypertension, left ventricle ejection fraction, and the Norton score. Gender (prespecified) was also included in the model. Furthermore, to adjust for differences in patient characteristics, propensity score‐matching was performed. Propensity scores were estimated using a multivariate logistic regression model. A local optimal algorithm with the caliper method was used for the development of propensity score‐matched pairs without replacement (1:1 match). A matching caliper of 0.2 standard deviations of the logit of the estimated propensity score was enforced to ensure that matches of poor fit were excluded. After propensity score‐matching, covariates were compared as described previously.

To evaluate the ability of a Norton scale score to predict all‐cause mortality, we estimated receiver operating characteristic (ROC) curves and area under the curve (AUC) with a 95% confidence interval (CI) using corresponding logistic models. Furthermore, to predict the benefit incurred by the addition of a Norton scale score to a baseline model of mortality prediction, we estimated net reclassification improvement (NRI). Using binary logistic regression, we computed the predicted risk for 10‐year mortality from a baseline model without a Norton score (age, gender, diabetes mellitus, hypertension, obesity, anemia, renal impairment, peripheral vascular disease, pulmonary hypertension, left ventricular ejection fraction, New York Heart Association [NYHA] function class) and a similar model that included a Norton score.

Statistical significance was assumed when the null hypothesis could be rejected at *p* < .05. All *p* values were the results of two‐sided tests. Statistical analyses were conducted using R (version 3.4.1).

## 3 RESULTS

During the 17‐year study period, 1469 patients underwent isolated AVR for the first time and were included in the study: 56% were males, and the mean age was 68 ± 13 years. The study population had a high burden of comorbidities: 71% had hypertension, 63% hyperlipidemia, and a third presented with diabetes mellitus, obesity, or smoking history. Most patients had AV stenosis (85%) and unicuspid or bicuspid AV was found in 35% of the patients.

### 3.1 Baseline characteristics by low (<18) and high (≥18) Norton score

There were 618 patients (42%) with a low, and 851 patients (58%) with a high Norton score. Baseline characteristics are shown in Table 1. While low Norton‐score patients were older, with a female predominance, and had a higher prevalence of comorbidities: hypertension, diabetes, chronic renal failure, and anemia, they were noted to have less unicuspid or bicuspid AV (Table 1). No significant difference was found in patients' body mass index, previous percutaneous coronary intervention (PCI), history of atrial fibrillation, COPD, prior cerebrovascular events, or left ventricular ejection fraction.

**Table 1:** Patient characteristics

	Norton score <18 (*N* = 618)	Norton score ≥18 (*N* = 851)	*p* Value
Age in years (mean ± SD)	69.6 ± 12.5	66.4 ± 13.1	<.001
Gender (male) (%)	283 (45.8)	536 (63)	<.001
BMI (mean ± SD)	28.3 ± 5.3	28.4 ± 5	.651
Obesity (%)	212 (34.4)	250 (29.4)	.050
Hypertension (%)	458 (74.4)	583 (68.6)	.019
PVD (%)	47 (7.7)	43 (5.1)	.061
Diabetes mellitus (%)	220 (35.7)	259 (30.5)	.040
Previous PCI (%)	125 (20.2)	162 (19)	.616
Previous MI (%)	55 (8.9)	77 (9)	.995
Atrial fibrillation (%)	57 (9.4)	70 (8.2)	.505
Hyperlipidemia (%)	375 (61)	553 (65.1)	.122
Family history of CAD (%)	66 (11.3)	110 (13.4)	.270
Smoking (%)			.995
Never	420 (68.6)	578 (68.8)	
Past smoker	110 (18)	151 (18)	
Current smoker	82 (13.4)	111 (13.2)	
COPD (%)	61 (10)	68 (8)	.227
Chronic renal failure (%)	87 (16)	87 (11)	.010
Prior CVA/TIA (%)	59 (9.7)	77 (9.1)	.805
Neurological deficit (%)	17 (4.5)	19 (3.6)	.598
Hypothyroid (%)	30 (5.9)	42 (5.8)	1.000
Systolic PAP ≥ 60 mmHg (%)	37 (6.2)	33 (4)	.066
NYHA functional class (%)			<.001
I	67 (11.4)	127 (15.4)	
II	262 (44.5)	429 (52)	
III	230 (39)	253 (30.7)	
IV	30 (5.1)	16 (1.9)	
Ejection fraction (%)	55.5 ± 10.9	55.8 ± 10.5	.560
Euroscore (standard) (mean ± SD)	6.6 ± 3	5.5 ± 2.5	<.001
Euroscore (logistic) (mean ± SD)	8.9 ± 8.9	6.2 ± 6	<.001
Hemoglobin level (mean ± SD)	12.3 ± 1.8	12.8 ± 1.7	<.001
Aortic valve lesion (%)			.001
Aortic stenosis	449 (78.7)	546 (70.7)	
Aortic insufficiency	79 (13.8)	123 (15.9)	
Both	43 (7.5)	103 (13.4)	
Aortic valve leaflets (%)			<.001
Unicuspid	5 (1.1)	23 (3.6)	
Bicuspid	131 (27.6)	225 (35.2)	
Tricuspid	338 (71.3)	392 (61.2)	

Abbreviations: CAD, coronary artery disease; COPD, chronic obstruction pulmonary disease; CVA, cerebral vascular accident; MI, myocardial infarction; NYHA, New York Heart Association; PAP, pulmonary artery pressure; PCI, percutaneous coronary intervention; PVD, peripheral vascular disease; SD, standard deviation; TIA, transient ischemic attack.

### 3.2 Surgical procedures

A median sternotomy approach was performed in 1197 patients (87.2%); a partial sternotomy (J‐sternotomy) was performed in 147 patients (10.7%), and a right mini‐thoracotomy in 29 patients (2.1%): 1205 patients (85%) received a biological prosthesis and 213 (15%) a mechanical prosthesis. Operative data were similar between the groups, with the exception of the median implanted prosthetic valve size that was smaller in the low Norton score group (Table 2).

**Table 2:** Operative data

	Norton score <18 (*N* = 618)	Norton score ≥18 (*N* = 851)	*p* Value
Minimally invasive (%)			.359
Mid‐sternotomy	511 (88.4)	686 (86.3)	
J‐sternotomy	58 (10)	89 (11.2)	
Right mini‐thoracotomy	9 (1.6)	20 (2.5)	
Prosthetic type (%)			.106
Biological	516 (86.9)	689 (83.6)	
Mechanical	78 (13.1)	135 (16.4)	
Prosthetic size (median [IQR])	21 [21–23]	23 [21–25]	<.001
Cross‐clamp time (min)	65.3 ± 21.1	65.3 ± 20.1	.983
Cardiopulmonary bypass time (min)	89.9 ± 32.8	89.2 ± 32.3	.680
Total operative time (min)	244 ± 62.4	241.5 ± 59.3	.462

Abbreviation: IQR, interquartile range.

### 3.3 In‐hospital outcomes by Norton score

**Figure 1:**
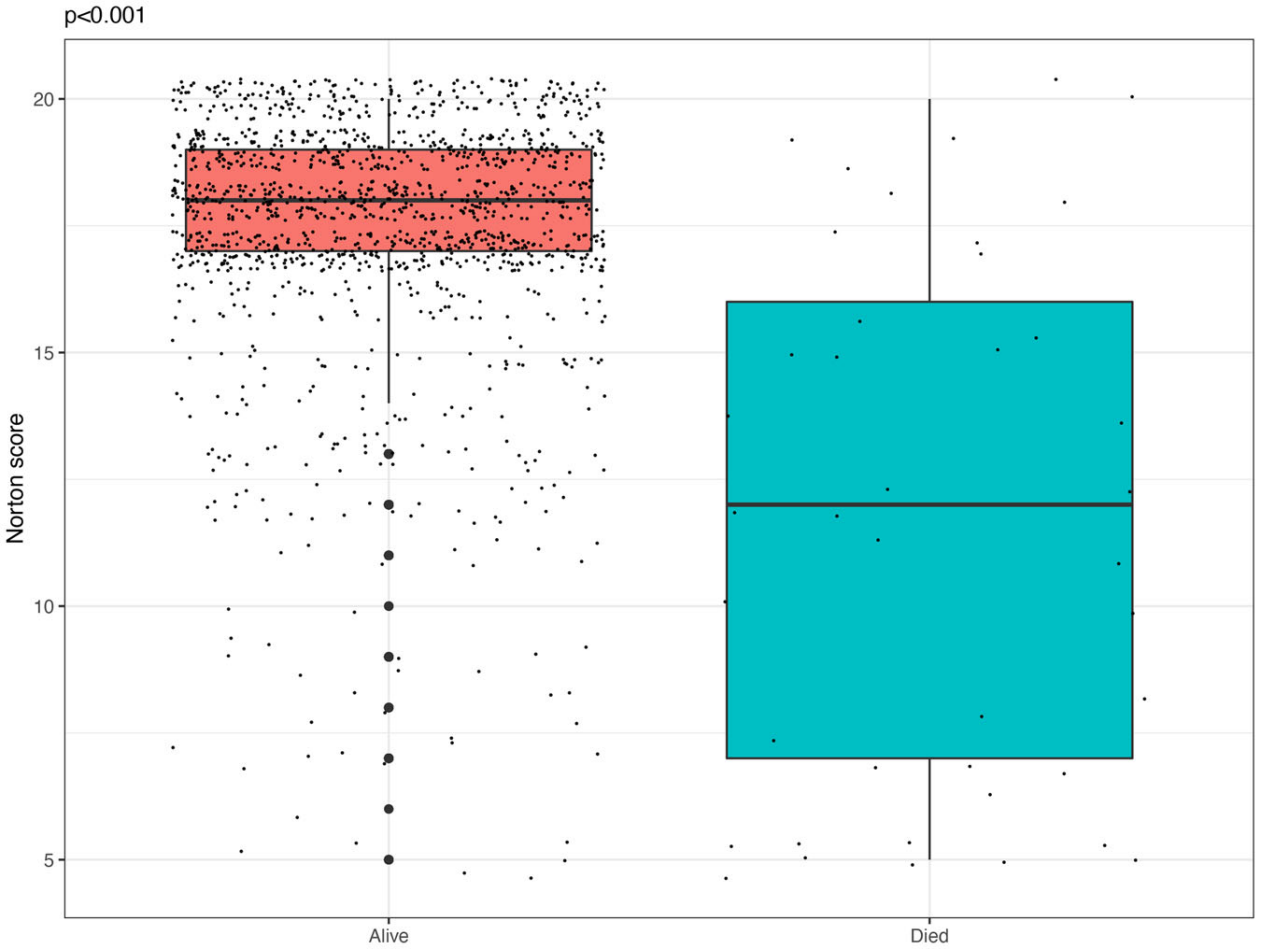
Boxplot of Norton score of patients who survived and died during hospitalization after aortic valve replacement. Each dot represents a different patient from the entire cohort. The mean Norton score of patients who survived was 17.5 ± 2.4 and mean Norton score of patients who died during hospitalization was 11.5 ± 5.2 (*p* < .001).

Univariable comparison demonstrated higher in‐hospital mortality among frailer patients with a low compared with a high Norton score (5.5% vs. 0.8%, *p* < .001). The Norton score was significantly higher among patients who survived compared to those who died after their AVR (17.5 ± 2.4 vs. 11.5 ± 5.2, *p* < .001) (Figure 1). Consistent with these findings, a multivariable logistic regression model (Supporting Information: Figure S1) showed that a low Norton score was associated with a threefold increased risk of in‐hospital mortality (odds ratio [OR] 3.03; 95% CI 1.14–0.09, *p* = .034). Furthermore, multivariate analysis, including the Norton score as a continuous measure, showed that each one‐point decrement in the Norton score was independently associated with a significant 28% increased risk for in‐hospital mortality (OR 1.28; 95% CI 1.14–1.43, *p* < .001).

**Figure 2:**
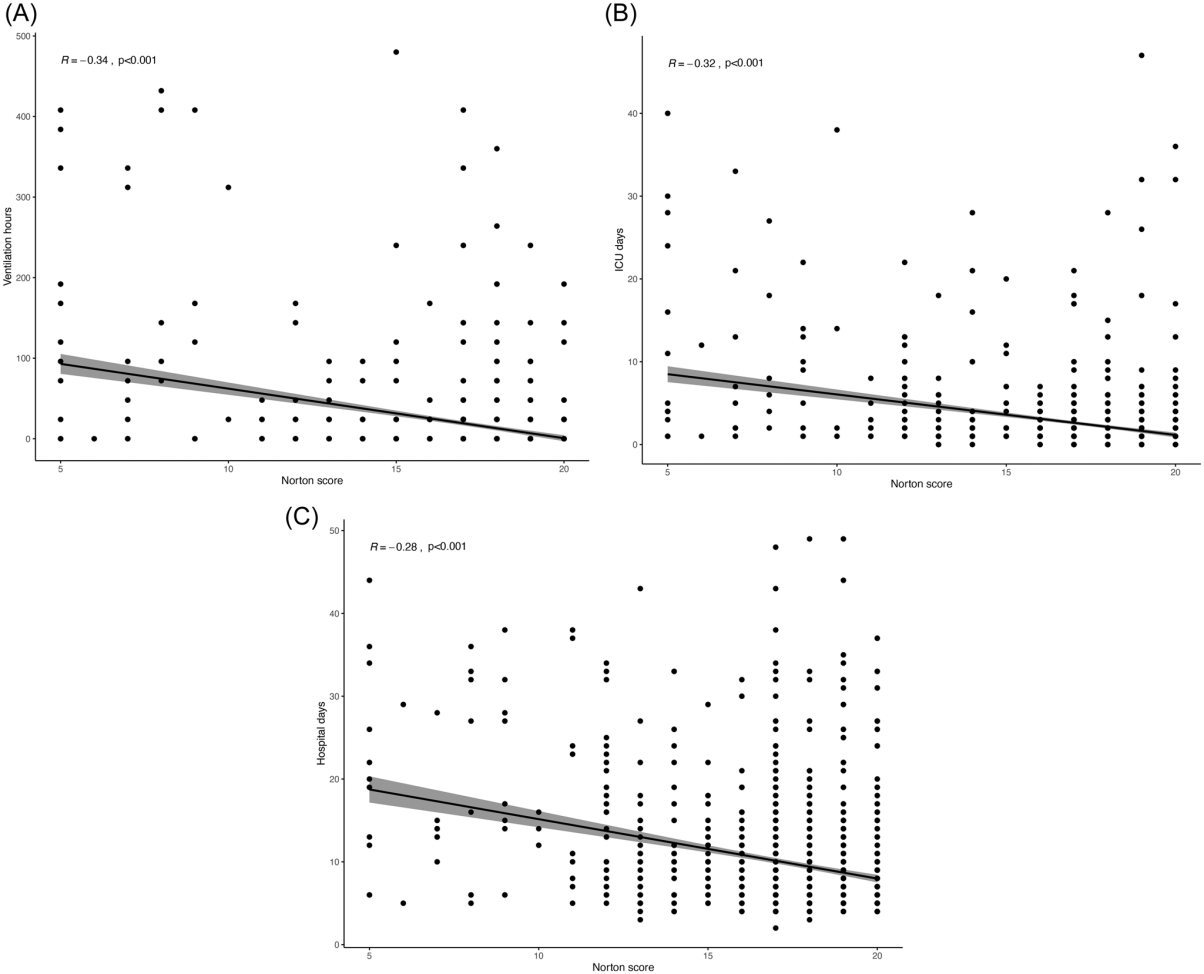
(A) Scatter plot of ventilation time in hours by Norton score. The statistical method used was the Pearson correlation. (B) Scatter plot of intensive care unit stay in days by Norton score. The statistical method used was the Pearson correlation. (C) Scatter plot of hospitalization time in days by Norton score. The statistical method used was the Pearson correlation.

Frailer patients with a low Norton score experienced longer ventilation time (24.2 ± 2.7 vs. 12.2 ± 1.3 h, *p* < .001), ICU stay (3 ± 4.7 vs. 2.1 ± 3.4 days, *p* < .001), hospitalization time (11.4 ± 8.6 vs. 9.3 ± 6.9 days, *p* < .001) (Figure 2A–C), and more acute kidney injury (11.5% vs. 6.5%, *p* = .001), compared to patients with a high Norton score.

### 3.4 Long‐term mortality by frailty

**Figure 3:**
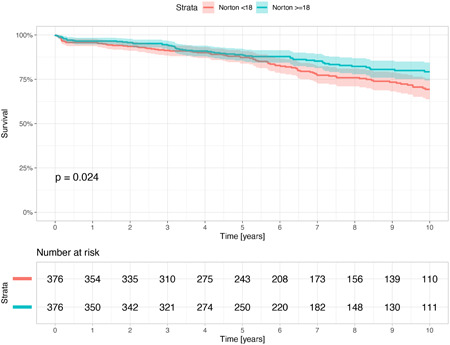
Kaplan–Mayer curves for survival at 10 years after propensity score‐matching by the low and high Norton score groups.

Kaplan–Meier survival analysis demonstrated long‐term (10‐year) higher mortality among frailer patients with a low compared with a high Norton score. The low Norton group had twice as much mortality compared with the high Norton group at 10 years of follow up (25.9%, 13.3%, log‐rank *p* < .001) (Supporting Information: Figure S2). Furthermore, also among the 752 matched patients (Supporting Information: Table S2), survival probability was higher among patients with high Norton score (Figure 3). Consistent with these findings, multivariable analysis showed that a high Norton score in the entire cohort was independently associated with a significant 37% reduction in the risk of 10‐year mortality compared with a low Norton score (95% CI 0.48–0.82, *p* < .001). Additional predictors of 10‐year mortality included older age (HR 1.06, 95% CI 1.04–1.07, *p* < .001), peripheral vascular disease (HR 1.75, 95% CI 1.17–2.63, *p* = .010), diabetes mellitus (HR 1.61, 95% CI 1.24–2.09, *p* < .001), renal impairment (HR 1.47, 95% CI 1.07–2.02, *p* = .020), and pulmonary hypertension (HR 2.99, 95% CI 2.02–4.41, *p* < .001).

### 3.5 Predictive ability of the Norton model

**Figure 4:**
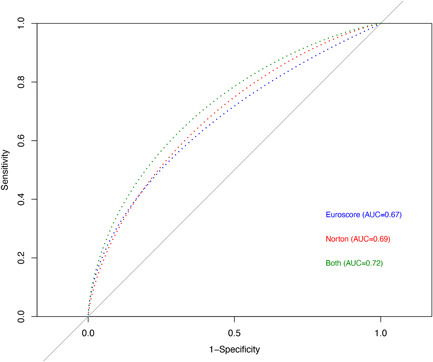
Receiver operating characteristic curve of admission Norton scale scores, logistic EuroScore and combination of Norton score and logistic EuroScore predicting 10‐year mortality. AUC, area under the curve.

The AUC of the Norton score (continuous) yielded 0.69 (95% CI 0.65–0.72) in predicting 10‐year mortality following AVR—comparable to the logistic EuroScore (AUC 0.67, 95% CI 0.63–0.71) (*p* = .474). However, a combination of the Norton score and the logistic EuroScore significantly increased (*p* < .001) the mortality predictive value compared to each of the scores separately (AUC 0.72, 95% CI 0.68–0.75) (Figure 4).

The addition of the Norton score to standard prognostic factors (age, gender, diabetes mellitus, hypertension, obesity, anemia, chronic kidney disease, peripheral vascular disease, pulmonary hypertension, left ventricular ejection fraction, NYHA function class) has shown a significant NRI of 59.4% (95% CI 37.9–80.8%, *p* < .001) for predicting 1‐year mortality, and an NRI of 40.6% (95% CI 27.7–53.5%, *p* < .001) for predicting 10‐year mortality.

## 4 DISCUSSION

This study, carried out in a contemporary cohort of patients who underwent their first isolated AVR, demonstrates several important implications regarding the impact of frailty on early outcomes and long‐term mortality. We have shown that (1) Norton score is a powerful predictor of clinical outcomes among patients who undergo AVR and a predictor of short‐ and long‐term mortality following AVR. Notably, separation in event rates was more pronounced immediately after surgery and continued thereafter, suggesting that the Norton score is a reliable marker identifying a high‐risk population prone to adverse events; (2) the reliability of the Norton score as a prognostic marker was maintained when used as a continuous measure, with each one point decrement in the scale corresponding to a significant 28% increase in‐hospital mortality risk and a 16% increase at 10 years; (3) the Norton score was found to be an independent predictor of mortality after adjustments to other risk factors with the added value of identifying patients at high risk, when combined with known prognostic factors.

Frailty is a syndrome that reflects a state of decreased physiological reserve.^7^, ^8^ Following surgery, frail patients are at marked risk for clinical decompensation, procedural complications, prolonged recovery, functional decline, disability, and mortality.^9^ The biological context of frailty includes systemic dysregulation of the immune, hormonal, and endocrine systems resulting in upregulation of inflammatory cytokines, and insulin resistance.^10^, ^11^, ^12^, ^13^, ^14^, ^15^ The subsequent catabolic state that ensues precipitates a progressive decline in muscle mass and strength.^16^ Medical practitioners estimate every patient for frailty. In most cases, this frailty estimation is subjective. We feel that an objective frailty estimation tool can benefit.

Most tools used to measure frailty focus on 1 or more of the 5 core domains that define the frailty phenotype: slowness, weakness, low physical activity, exhaustion, and shrinking.^17^ Most of these phenotypes are difficult to measure. On the other hand, Norton scale scores are simple to measure and, accordingly, can be used as a screening assessment tool by nurses and physicians. Furthermore, the Norton scoring system is not time‐consuming.

AVR and TAVI are the only effective treatments for severe AS. Currently, however, TAVI is limited to moderate and high‐risk patients only, when the risk of TAVI is estimated to be lower than the risk of AVR, taking into consideration the fact that long‐term results of TAVI are still unknown.^18^ Both the Society of Thoracic Surgeons (STS) score and the EuroSCORE II were validated to predict 30‐day mortality after cardiac surgery.^19^, ^20^ In the current guidelines, recommendations for selecting an intervention mode between AVR and TAVI are based on these risk stratification tools. Surgical AVR is recommended in patients at low surgical risk (STS or EuroSCORE II < 4%) with no other risk factors that are not included in these scores, such as porcelain aorta, sequelae of chest radiation, or frailty (class IB).^21^ However, no specific tool to measure frailty is mentioned. Our findings show that the admission Norton score is a powerful marker of mortality, and, therefore, should be considered as a risk stratification tool in patients who are candidates for AVR or TAVI.

In a previous publication by Afilalo et al., frailty was shown to be a significant risk factor for death and disability following AVR and TAVI using a four‐item scale that include lower‐extremity weakness, cognitive impairment, anemia, and hypoalbuminemia. At their study, outcomes of interest were all‐cause mortality and disability 1 year after the procedure.^22^ Another publication using the Norton score as a frailty scale and mortality prediction tool following TAVI, also reported on 1‐year mortality.^5^ Our report is the first that shows the ability of the Norton score to predict long‐term mortality following AVR.

This report lays the foundation for future studies regarding preoperative rehabilitation intervention for older and frail patients who are selected to undergo elective cardiac procedures, to improve their physical performance before surgery.

### 4.1 Limitations

There are a few limitations in our study. First, despite it being retrospective in design, data were collected prospectively and recorded in a well‐defined database. Second, our study was conducted in a single‐center cardiac surgery department. Third, we had no information regarding the main cause of death, the rate of cardiac events, and data regarding prosthetic valve performance during the follow‐up period. The lack of information regarding the main cause of death weakens the conclusions of this study.

## 5 CONCLUSIONS

Frailty is a major predictor of short‐ and long‐term mortality and should be taken into account when assessing patients considered suitable to undergo AVR. The Norton admission score is an objective tool to estimate patients' frailty. It can be used to identify patients prone to adverse outcomes after AVR.

## CONFLICT OF INTEREST

The authors declare no conflict of interest.

## Supporting information

Supporting information.

Supporting information.

Supporting information.

Supporting information.
