# Supplementary figures and images for: Frailty and clinical outcomes following aortic valve replacement
Source: J Card Surg. 2022 Aug 3;37(10):3036–43. doi: 10.1111/jocs.16801 (PMC9544524; doi:10.1111/jocs.16801)

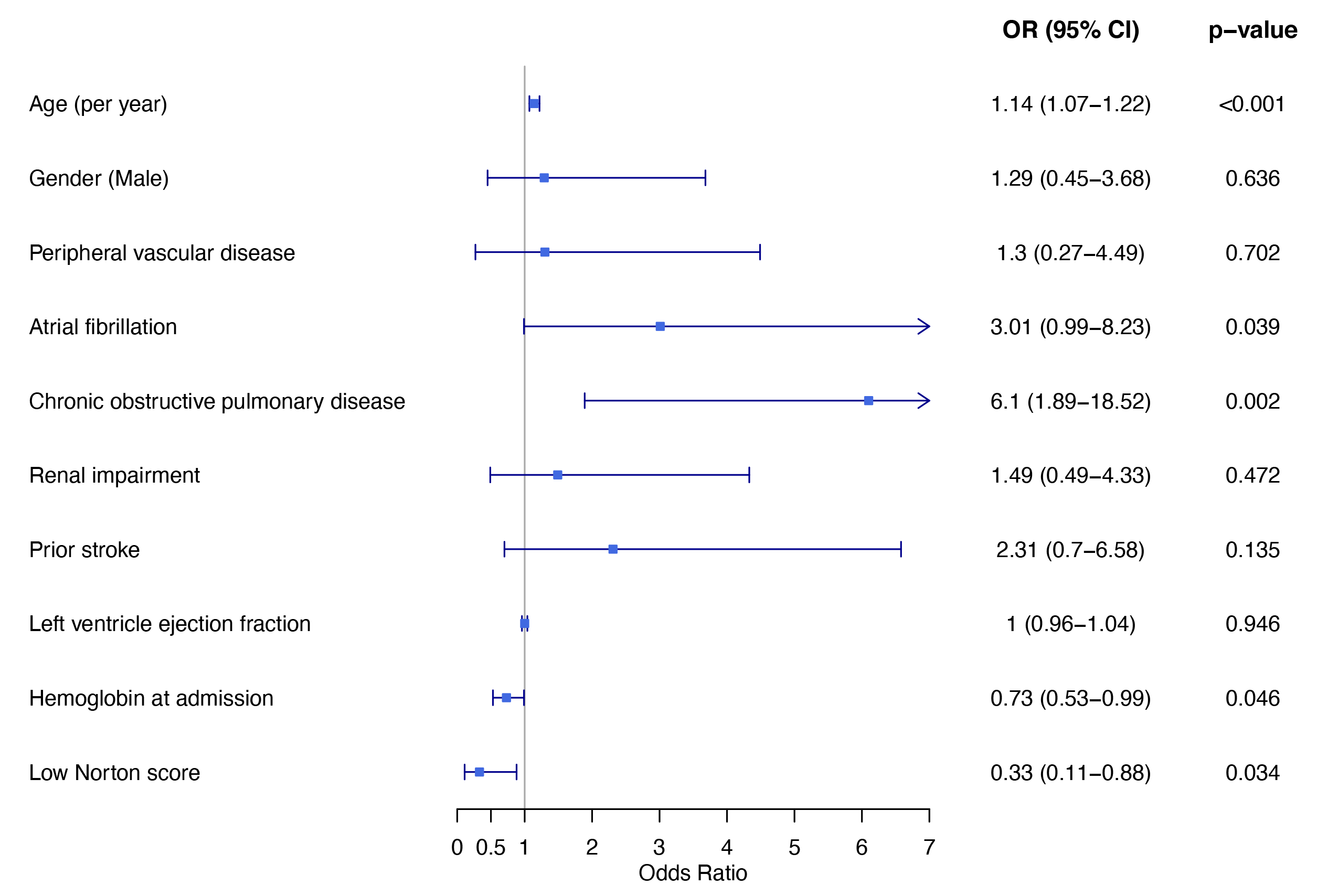

Supplement: Supplementary file 1 — Supporting information. [file JOCS-37-3036-s001.png]

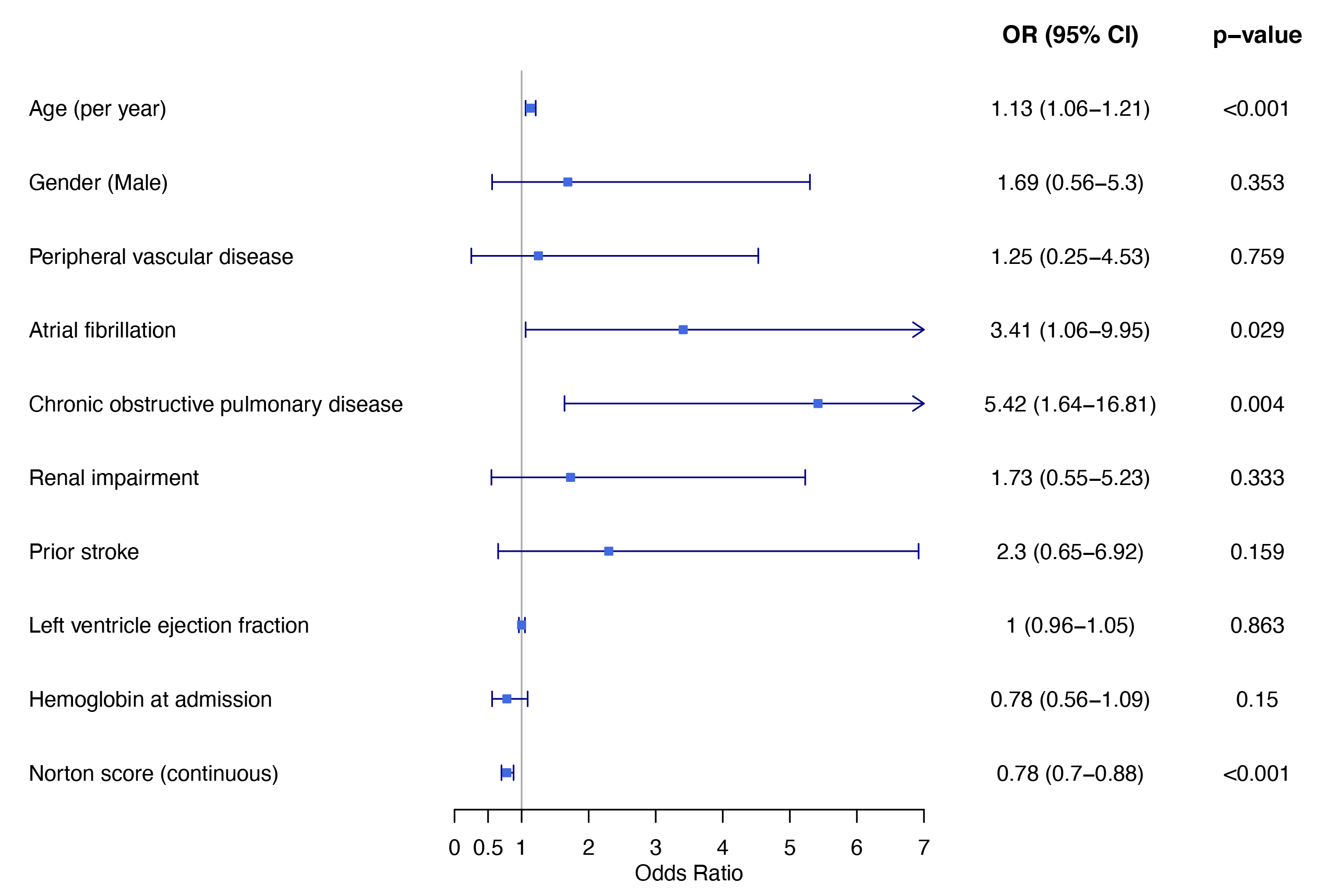

Supplement: Supplementary file 2 — Supporting information. [file JOCS-37-3036-s004.png]

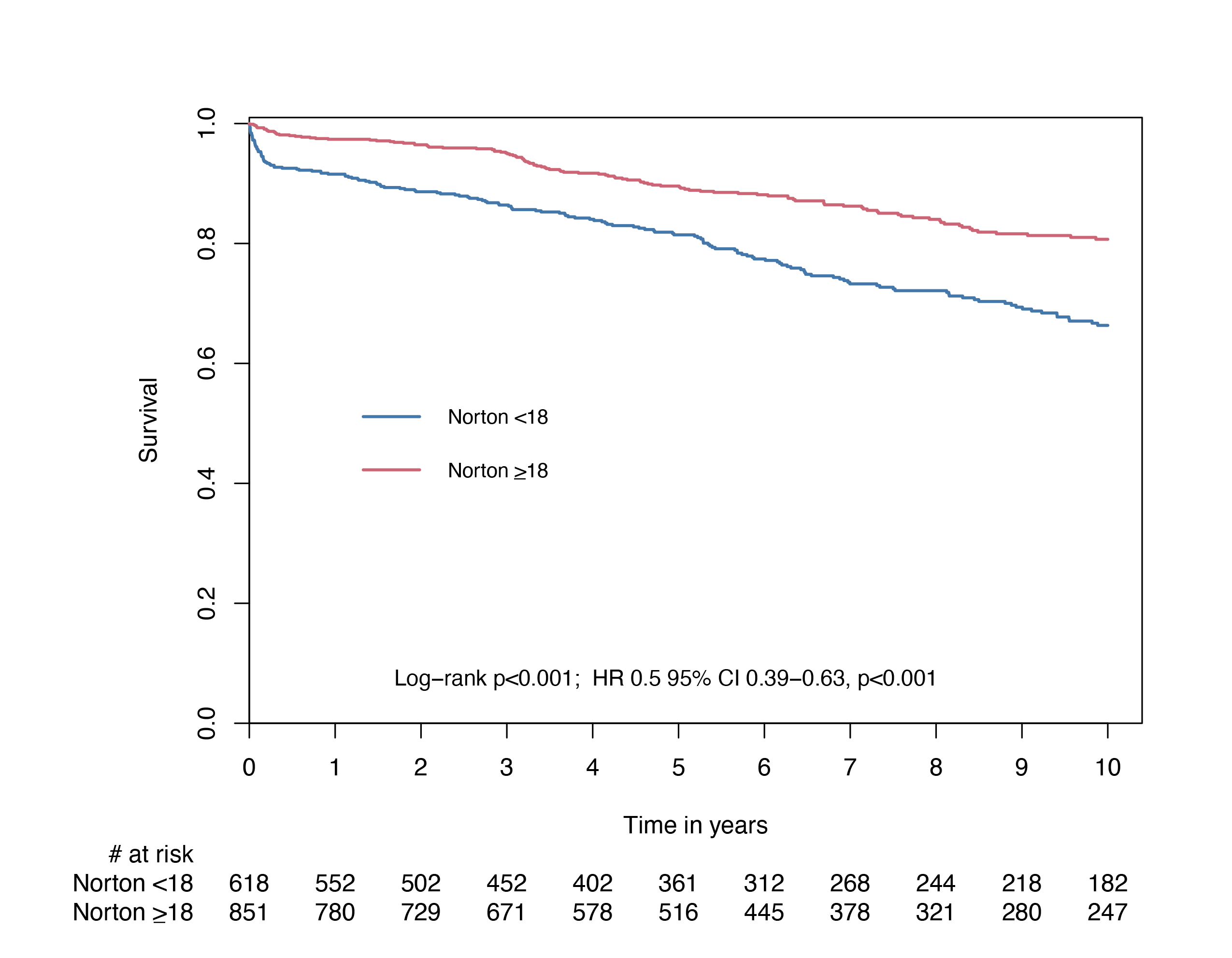

Supplement: Supplementary file 3 — Supporting information. [file JOCS-37-3036-s003.png]
