# Supplementary material for: Frailty and clinical outcomes following aortic valve replacement
Source: J Card Surg. 2022 Aug 3;37(10):3036–43. doi: 10.1111/jocs.16801 (PMC9544524; doi:10.1111/jocs.16801)
Supplement: Supplementary file 4 — Supporting information. [file JOCS-37-3036-s002.docx]

**Supplemental Appendix**

**Table S1:** Norton scoring system

| **Domains** | **Scores** |
| --- | --- |
| Physical condition | 4 = Good |
|  | 3 = Fair |
|  | 2 = Poor |
|  | 1 = Very bad |
|  |  |
| Mental condition | 4 = Alert |
|  | 3 = Apathetic |
|  | 2 = Confused |
|  | 1 = Stuporous |
|  |  |
| Activity (in daily living) | 4 = Independent |
|  | 3 = Slightly dependent  2 = Very dependent |
|  | 1 = Fully dependent |
|  |  |
| Mobility | 4 = Ambulant |
|  | 3 = Walks with help  2 = Chair bound |
|  | 1 = Bedridden |
|  |  |
| Incontinence | 4 = None |
|  | 3 = Occasional |
|  | 2 = Usually urinary |
|  | 1 = Urinary and fecal |

**Table S2: Patient characteristics after propensity score matching**

|  | Norton score <18  (N=376) | Norton score ≥18 (N=376) | p-value | SMD |
| --- | --- | --- | --- | --- |
| Age in years (mean±SD) | 68.6 **±** 12.7 | 68.7 **±** 11.6 | 0.902 | 0.009 |
| Gender (male) (%) | 191 (50.8) | 193 (51.3) | 0.942 | 0.011 |
| BMI (mean±SD) | 28.5 **±** 5.3 | 28.8 **±** 5.1 | 0.383 | 0.065 |
| Obesity (%) | 107 (28.5) | 106 (28.2) | 1.000 | 0.006 |
| Hypertension (%) | 270 (71.8) | 275 (73.1) | 0.744 | 0.030 |
| PVD (%) | 28 (7.4) | 26 (6.9) | 0.888 | 0.021 |
| Diabetes mellitus (%) | 135 (35.9) | 140 (37.2) | 0.762 | 0.028 |
| Previous PCI (%) | 83 (22.1) | 73 (19.4) | 0.418 | 0.066 |
| Previous MI (%) | 31 (8.2) | 35 (9.3) | 0.699 | 0.038 |
| Atrial fibrillation (%) | 34 (9.1) | 31 (8.2) | 0.787 | 0.029 |
| Hyperlipidemia (%) | 243 (64.6) | 248 (66) | 0.759 | 0.028 |
| Family history of CAD (%) | 43 (11.5) | 39 (10.5) | 0.764 | 0.031 |
| Smoking (%) |  |  | 0.992 | 0.009 |
| Never | 253 (67.3) | 253 (67.3) |  |  |
| Past smoker | 71 (18.9) | 72 (19.1) |  |  |
| Current smoker | 52 (13.8) | 51 (13.6) |  |  |
| COPD (%) | 29 (7.7) | 30 (8) | 1.000 | 0.010 |
| Chronic renal failure (%) | 47 (12.5) | 48 (12.8) | 1.000 | 0.008 |
| Prior CVA/TIA (%) | 38 (10.1) | 39 (10.4) | 1.000 | 0.009 |
| Neurological deficit (%) | 12 (5) | 7 (3.2) | 0.462 | 0.091 |
| Hypothyroid (%) | 21 (5.8) | 27 (7.5) | 0.449 | 0.068 |
| Systolic PAP ≥60 mmHg (%) | 16 (4.3) | 18 (4.8) | 0.861 | 0.026 |
| NYHA functional class (%) |  |  | 0.185 | 0.161 |
| I | 46 (12.2) | 43 (11.4) |  |  |
| II | 189 (50.3) | 187 (49.7) |  |  |
| III | 123 (32.7) | 138 (36.7) |  |  |
| IV | 18 (4.8) | 8 (2.1) |  |  |
| Ejection fraction (%) | 56.1 **±** 10.2 | 55.9 **±** 10.2 | 0.702 | 0.028 |
| Hemoglobin level (mean±SD) | 12.5 **±** 1.7 | 12.6 **±** 1.7 | 0.359 | 0.070 |

SMD = Standardized mean difference; SD = Standard deviation; BMI = Body mass index; PVD = Peripheral vascular disease; PCI = Percutaneous coronary intervention; MI = Myocardial infarction; CAD = Coronary artery disease; COPD = Chronic obstruction pulmonary disease; CVA = Cerebral vascular accident; TIA = Transient ischemic attack; PAP = Pulmonary artery pressure; NYHA = New York Heart Association.

**Figure S1:**

Multivariable logistic regression: OR for in-hospital mortality with 95% CI. Norton score is a covariate as a categorical variable (<18) (A) or continues measure (B).
OR = Odds ratio; CI = confidence interval.

**Figure S2:**

Hazard plot for survival at 10years by the low and high Norton score groups.

* The HR is for univariable Cox analysis

HR = Hazard ratio; CI = Confidence interval.
